# The Application of Multifunctional Metal–Organic Frameworks for the Detection, Adsorption, and Degradation of Contaminants in an Aquatic Environment

**DOI:** 10.3390/molecules30061336

**Published:** 2025-03-17

**Authors:** Yachen Liu, Jinbin Yang, Junlin Wu, Zehao Jiang, Xinyu Zhang, Fanjun Meng

**Affiliations:** 1 School of Chemistry, Chemical Engineering and Materials Science, Shandong Normal University, Jinan 250014, China; 2024319025@stu.sdnu.edu.cn (Y.L.); 202310300107@stu.sdnu.edu.cn (J.W.); 202310100309@stu.sdnu.edu.cn (Z.J.); 19710156217@139.com (X.Z.); 2 Shandong High-Tech Medical Device Innovation Center Co., Ltd., Zibo 255000, China; yangjinbin@shinva.com

**Keywords:** multifunctional MOFs, detection, adsorption, degradation, wastewater treatment

## Abstract

Water pollution poses a severe threat to both aquatic ecosystems and human health, highlighting the crucial importance of monitoring and regulating its levels in water bodies. In contrast to traditional single-treatment approaches, multiple-treatment methods enable the simultaneous detection and removal of water pollutants using a single material. This innovation not only offers convenience but also fosters a more holistic and effective approach to water remediation. Metal–organic frameworks (MOFs) are versatile porous materials that offer significant potential for use in wastewater treatment. This article examines the latest developments in the application of MOFs for multifaceted wastewater treatment. MOFs are used for simultaneous detection and removal, or for the detection and degradation of contaminants. Some MOFs exhibited different functions for different contaminants, and some MOFs showed one function (adsorption or detection) for more than one contaminant. All the multifunctional MOFs facilitate the multiple treatment of the real wastewater. Lastly, existing challenges and future outlooks concerning MOF materials for wastewater treatment are also addressed in this paper.

## 1. Introduction

In recent decades, rapid advancements in science, technology, industry, and population growth have led to increasingly severe pollution in aquatic environments, becoming a global issue of growing concern [1,2]. The uncontrolled discharge of pollutants, including industrial wastewater, agricultural runoff, domestic sewage, and microplastics, not only disrupts aquatic ecosystems but also poses significant risks to human drinking water safety, thereby impacting public health [2,3]. As a result, water pollution has garnered widespread attention, prompting researchers to develop various methods for treating these contaminants.

To date, numerous wastewater treatment methods have been employed to restore or facilitate the restoration of aquatic environments [1,4,5], and they can largely be categorized into three strategies: sensing, separation, and degradation.

(1) Sensing: This method involves the detection of pollutants with high sensitivity and selectivity. Analytical techniques commonly used for detection include gas chromatography (GC), liquid chromatography (LC), Raman spectroscopy (RS), thin-layer chromatography (TLC), paper-based microfluidics, and electrochemical biosensing [4,6,7]. Additionally, various materials such as nanomaterials, porous materials, polymeric materials, and small molecules are developed to detect pollutants, often with the aid of fluorescence spectrometry, UV–visible spectrophotometry, or even visual inspection [8,9,10,11].

(2) Separation: Separation methods are commonly used to remove pollutants from water, including adsorption, membrane filtration, ion exchange, coagulation, chemical precipitation, flocculation, and electrodialysis [12,13,14]. Among these, adsorption-based removal is one of the most widely employed techniques for wastewater treatment [15,16,17,18,19]. A variety of materials with high adsorption capacity and efficiency, for instance zeolites, clay minerals, activated carbon, and other porous materials, have gained attention because of their low cost, environmental friendliness, and ease of use [17,20].

(3) Degradation: Degradation methods are widely used to break down organic pollutants in water, typically through oxidation or reduction reactions [21,22]. Several degradation techniques have been developed, including photocatalysis, electrocatalysis, the Fenton reaction and Fenton-like reactions, ozonation, and chlorination [22,23,24,25]. Photocatalysis, which utilizes solar energy to generate photogenerated carriers (electrons and holes) on semiconductors, is a particularly promising approach [26]. Over time, various semiconductors, including metal oxides (e.g., TiO_2_, Fe_2_O_3_, ZnO), metal sulfides (e.g., CuS, CdS, FeS), and high-entropy alloys, have been developed [4,27,28]. Additionally, organic materials such as g-C_3_N_4_, graphene, carbon nanotubes, covalent organic frameworks (COFs), and conjugated polymers have been explored as photocatalysts [28,29,30,31,32]. Inorganic–organic hybrid materials, such as metal–organic frameworks (MOFs) and metal–organic complexes, are also commonly used [31,33]. Recently, MOFs have garnered considerable interest owing to their distinctive characteristics, showing great promise in wastewater treatment [20,34].

In the development of materials for wastewater treatment, single-functional materials have traditionally been the focus of research. For example, materials designed for pollutant sensing typically have limited separation or degradation capabilities [2,20]. Similarly, materials used for separation generally do not possess strong sensing or degradation abilities. While materials with both separation and degradation functions are sometimes combined for pollutant treatment, it remains a significant challenge to integrate multiple functions into a single catalyst [20,35]. This is due to the difficulty in matching components with high compatibility while minimizing the interference between them [36]. Nonetheless, researchers have been working on the development of multifunctional materials due to their promising potential.

For instance, a sensing probe with separation capabilities allows pollutants to be detected and subsequently separated without concerns of probe contamination, as it can continue to function in subsequent treatment steps [6,9]. Similarly, a semiconductor catalyst used in photodegradation benefits from enhanced performance if it also has a strong adsorption capacity, leading to improved treatment efficiency [37]. Additionally, a porous material used for pollutant adsorption may not require regeneration (typically achieved by desorption) if it also possesses degradation functionality [4,37]. Consequently, multifunctional materials are receiving increasing attention and are being developed across diverse fields, such as biomedicine, energy, and materials science.

Moreover, because real-world wastewater is often contaminated with a wide variety of pollutants, it is inefficient and economically unfeasible to treat only one type of contaminant at a time [20]. To address this, many researchers have developed multifunctional materials capable of simultaneously treating multiple pollutants. For example, a sensing probe may detect several pollutants at once [38,39], or a material may both adsorb and degrade different contaminants simultaneously [4,40]. The ability to treat multiple pollutants with a single material or process represents a significant advancement in wastewater treatment. Therefore, investigating materials with multiple functions, such as sensing, separation, or degradation, or materials capable of addressing multiple pollutants at once is of great importance [2,20]. However, the design and versatility required to construct such multifunctional materials remain a challenge.

Metal–organic frameworks (MOFs), crystalline materials made from organic linkers and metal nodes (e.g., metal clusters or ions), have garnered considerable attention because of their diverse geometries, ultra-high specific surface areas, and tunable structures and functionalities [3,33,41]. These features make MOFs highly suitable for applications in sensors, catalysis, energy storage, and adsorption [20,33,42]. Recently, MOFs have been increasingly explored in wastewater treatment, specifically for contaminant sensing, separation, and degradation.

MOF materials have demonstrated remarkable efficacy in wastewater treatment. Their porosity and specific adsorption sites endow them with a high adsorption capacity and efficiency for various contaminants in water [43,44]. Thanks to their tunable properties, MOFs can be modified with diverse ligands, metal ions, or clusters, thereby imparting them with sensing and catalytic functionalities [2,35]. Numerous studies have showcased MOFs’ diverse and exceptional capabilities, including high removal efficiency, low detection limits, and high catalytic activity in wastewater treatment [2,43]. When compared to other materials, the porosity and tunability of MOFs stand out prominently, granting them numerous advantages across different applications.

MOF materials exhibit significant and broad application prospects in numerous fields [43,44]. However, their large-scale industrial application still faces numerous challenges [45,46]. For instance, the stability of MOFs, which is susceptible to environmental factors such as temperature and pH values, is inferior to that of carbon materials and zeolites. Additionally, the preparation cost of MOFs poses a limitation on their widespread adoption. Furthermore, the challenge lies in skillfully constructing their structure to maximize their functional performance. Fortunately, researchers are diligently striving to overcome these obstacles and advance the application of MOFs [45].

While several reviews have addressed the use of MOFs in various applications, most focus on single-function MOFs [20,35]. Bu and colleagues [20] have reviewed the design of multifunctional MOFs and their application in water pollution treatment. However, the advancements in multifunctional MOFs over the past two years have yet to be comprehensively summarized. Moreover, only a few studies have explored MOFs that can treat more than one contaminant simultaneously.

**Figure 1:**
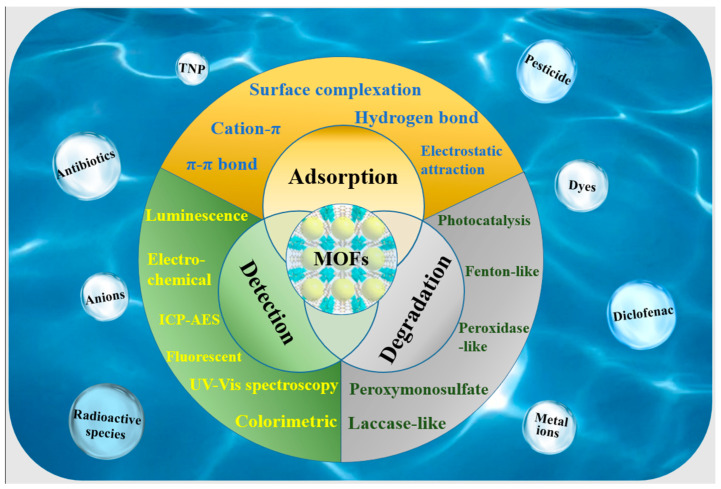
Treatment of various pollutants by multifunctional MOFs.

In this review, we examine the design and application of multifunctional MOF materials in sensing, separation (adsorption), and degradation, either for a single pollutant or for multiple pollutants, as shown in Figure 1. We also address the challenges associated with treating multiple water pollutants and propose strategies for future developments in this field.

## 2. Simultaneous Detection and Adsorption-Based Removal Contaminants

Due to their high porosity and specific adsorption sites on the surface, MOFs are often initially considered for their adsorption capabilities [17,47,48,49]. In addition, MOFs can be functionalized with specific ligands or metal ions, making them excellent candidates for sensor fabrication [36,50]. As a result, many studies have explored the multifunctionality of MOFs in the detection and removal of contaminants, as shown in Table 1.

**Table 1:** Summary of MOFs for simultaneous detection and removal of contaminants.

Catalyst	Contaminant	Detection		Adsorption Capacity	Time for Equilibrium	Year	Ref.
		Method	LOD				
Zr-MOF	TC and OTC	Fluorescent	6.14 and 14.59 nM	262.46 and 267.92 mg/g	30–360 min	2024	[6]
Znq_2_@ZIF-8	TC	Fluorescent	0.13 μmol/L	377.02 mg/g	30–360 min	2024	[8]
*apt*-NiCoFe-MOF-74	TC, OTC, CTC, and DOX	Fluorescent	1.3, 1.4, 5.5, and 3.0 nM	97.4, 105.2, 115.6, and 114.7 mg/g	10 min	2024	[9]
		Colorimetric	6, 10, 49, and 2.8 nM				
Zr-MOF (BMAT_3_H_5_)	TC	Fluorescent	28 ± 0.012 nM	317.6 mg/g.	5 min	2025	[10]
Fe_3_O_4_@PDA@Eu-MOF	TC	Fluorescent	2 μg/L	144.9 mg/g	80 min	2023	[11]
Zn-MOF	OTC	Fluorescent	26.9 nM	5.8 mg/g	4 h	2023	[51]
Eu-CuBi_2_O_4_@ZIF-8	TC	Fluorescent	17 nM	377.07 mg/g	2 h	2022	[52]
Eu-MOF	TC	Fluorescent	3 nM	387.14 mg/g	500 min	2021	[53]
Eu/Zr-MOF	TC	Fluorescent	0.92 ng/mL	289 mg/g	150 min	2021	[54]
Cd-MOF	chloramphenicol (CHL)	Fluorescent	91 ppb	-	50 h	2020	[55]
Eu^3+^-MOF	Malachite green (MG)	Fluorescent	34.20 nM	97.64% based on 20 mg Eu^3+^-MOF in 10 mL MG (10 mg/L) under pH 7 at 35 °C in 120 min.	120 min	2024	[15]
	Leuco-malachite green (LMG)		1.98 nM	-	-		
Zr-Sti	2,4,6-trinitrophenyl phenol (TNP)	Fluorescent	0.68 μM (156 ppb)	ca. 78 mg/g	-	2023	[56]
Cd-MOF@ macroporous melamine foam	TNP	Luminescence	0.38 μM	456.16 mg/g	30 min	2022	[57]
Cu^+^-tpp@ZIF-8	*P*-arsanilic acid	Fluorescent	0.4 µg/L	303.0 mg/g	240 min	2022	[58]
Poly(DES)@MOF	Diclofenac	UV spectrophotometer	0.84 μg/mL	19.39 mg/g	2 h	2024	[59]
ZIF-8-on-Zn_2_@SA	Pesticide: thiophanate-methyl [1.2-α-(3-methoxycarbonyl-2-thioureido)benzene]	Fluorescent	0.14 μM	161.8 mg/g	~280 min	2022	[37]
kgd-M1@ACPs	Pesticide: 2,6-dichloro-4-nitroaniline	Fluorescent	0.09 µM	83.3 mg/g	240 min	2022	[60]
Zn2@ZIF-8@SA	Pesticide: quinclorac, 2,6-dichloro-4-nitroaniline, and thiabendazole	Fluorescent	0.08, 0.09, and 0.37 μM	142.1 mg/g	20 h	2022	[61]
UiO-66-NH_2_@AuNCs/ZIF-8	Hg^2+^	Fluorescent	0.42 ppb	129.9 mg/g	30 min	2024	[62]
Sm-MOF	Hg^2+^	Fluorescent	0.87 μM	0.97 μmol/4 mg	2 h	2021	[63]
Zn_2_(BDC)_2_(TzTz)_2_	Hg^2+^	Colorimetric	-	1428 mg/g	30 s–30 min	2021	[64]
Thioketone Al-MOF nanorods	Hg^2+^	Colorimetric	0.8 ppb	1110 mg/g	-	2020	[65]
Zr-Sti	Cr_2_O_7_^2−^	Fluorescent	0.73 μM (159 ppb)	43.38 mg/g	-	2023	[56]
Dyes⊂MOF-801	Cr_2_O_7_^2−^	Fluorescent	0.03 mM	83 mg/g	3 min	2019	[66]
HSB-W15-NS	Fe^3+^	Fluorescent	0.837 μM	250.81 mg/g	5 min	2024	[67]
Zn-MOF	Fe^2+^	UV-Vis spectroscopy	0.129 μM	208.7 mg/g	30 min	2023	[68]
	Pb^2+^		0.113 μM	192.6 mg/g			
	V^5+^		0.246 μM	203.6 mg/g			
MIL-101(Fe)	Fe^3+^	Fluorescent	1.8 μM	3.5 mM/g	180 min	2019	[69]
	Cu^2+^		1.6 μM	0.9 mM/g	180 min		
	Pb^2+^		5.2 μM	1.1 mM/g	180 min		
Fe_3_O_4_/MOF/L-cysteine	Cd^2+^	ICP-AES	10.6 ng/mL	248.24 mg/g	10 min	2018	[70]
		Fluorescent	0.94 ng/mL				
NH_2_-MIL-88(Fe)	As^5+^	Fluorescent	4.2 ppb	125 mg/g	24 h	2017	[71]
Tb-BTC	phosphorus	Fluorescent	2.97 μM	222.2 mg/g	30 min	2023	[72]
In(tcpp)	F^−^	Fluorescent	1.3 μg/L	36.7 mg/g	30 min	2021	[73]
	Perfluorooctanoic acid		1.9 μg/L	980.0 mg/g	30 min		
Ag@UiO-66-(COOH)_2_	I^−^	Fluorescent	0.58 ppm	235.5 mg/g	60 min	2022	[74]

### 2.1. Organics

#### 2.1.1. Antibiotic Treatment

Antibiotic contamination in wastewater has garnered significant attention due to its potentially detrimental impacts on ecosystems and human health. This complex issue requires comprehensive approaches for effective treatment [4,75,76]. Various MOF-based materials have been applied to address antibiotic pollution in aquatic environments.

**Figure 2:**
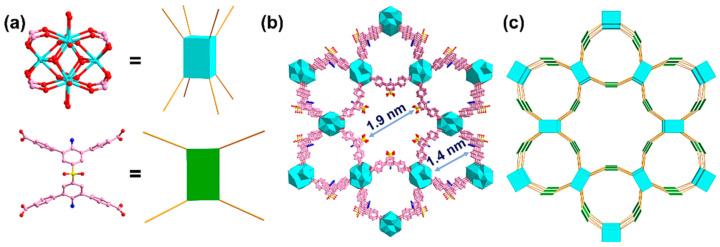
(**a**) The Zr_6_ cluster and H_4_SBTD-NH_2_ ligand. (**b**) The 3D framework of Zr-MOF. (**c**) (4,8)-connected *csq* net of Zr-MOF [6].

Li et al. [6] developed a dual-functional Zr-based MOF (Zr-MOF) using a tetra-carboxylate ligand, H_4_SBTD-NH_2_ (as shown in Figure 2), for the simultaneous detection and removal of tetracyclines (TCs). The detection was based on a luminescence quenching effect for both TC and oxytetracycline (OTC), with limits of detection (LOD) of 6.14 nM and 14.59 nM, respectively, showing good stability and selectivity. Zr-MOF also exhibited excellent adsorption capacities of 262.46 and 267.92 mg/g for TC and OTC, respectively. The detection and adsorption mechanisms were attributed to the inner filter effect (IFE), electron transfer, and specific host–guest interactions between Zr-MOF and the contaminants. The results of FT-IR spectra demonstrated that the hydrogen bond and π-π stacking between benzene rings of TCs and Zr-MOF contributed to the adsorption. Additionally, the Zr-O bond might also be involved in the adsorption process. This work highlights the potential of Zr-MOF as a multifunctional material for treating antibiotics in aquatic environments. The usage of ligands with various functional group is an ideal method for endowing MOFs with different functions, making them obviously superior to other materials, such as carbon materials and zeolites.

Yang and coworkers [8] synthesized a hybrid MOF material, Znq_2_@ZIF-8, with an octahedral core–shell structure by crystallizing ZIF-8 on the Znq_2_ surface. A strong green emission (495 nm) was observed for the Znq_2_@ZIF-8 solution. Upon the introduction of tetracycline (TC), the fluorescence was quenched due to energy competition absorption, indicating the material’s recognition ability for TC and structurally similar molecules. The selectivity for TC was confirmed through tests with various ions and antibiotics, with an LOD of 0.13 μmol/L. Additionally, Znq_2_@ZIF-8 exhibited good adsorption capacity for TC removal. It was found that π-π stacking between the TCs (benzene rings) and Znq_2_@ZIF-8 (imidazole rings and benzene rings), and coordination bonds between the Zn metal center and the amino and/or hydroxyl groups of TCs might be generated, which may have contributed to the adsorption. The study demonstrated its multifunctional potential for the simultaneous detection and removal of TC from wastewater.

Wang and Zhang [9] developed NiCoFe-MOF modified with TC aptamers (apt-NiCoFe-MOF-74), resulting in a controllable hollow structure. This material displayed both colorimetric and fluorescent detection capabilities, as well as excellent removal efficiency for TCs in water. Using a colorimetric detection system with 3,3,5,5-tetramethylbenzidine (TMB), H_2_O_2_, and NaAc-HAc buffer, very low LODs were achieved for tetracycline (TET), oxytetracycline (OTC), chlortetracycline (CTC), and doxycycline (DOX), at 6 nM, 10 nM, 49 nM, and 2.8 nM, respectively. In fluorescence detection, the LODs were 1.3, 1.4, 5.5, and 3.0 nM for TET, OTC, CTC, and DOX, respectively. The apt-NiCoFe-MOF-74 exhibited strong adsorption capacities for these antibiotics, with values of 97.4, 105.2, 115.6, and 114.7 mg/g for TET, OTC, CTC, and DOX, respectively. The material demonstrated its applicability in real sample detection, such as the analysis of TC contamination in honey. The modification of transition metals on MOFs is a common and effective method to endow MOFs with various functions, because those metals have an excellent catalytic ability.

**Figure 3:**
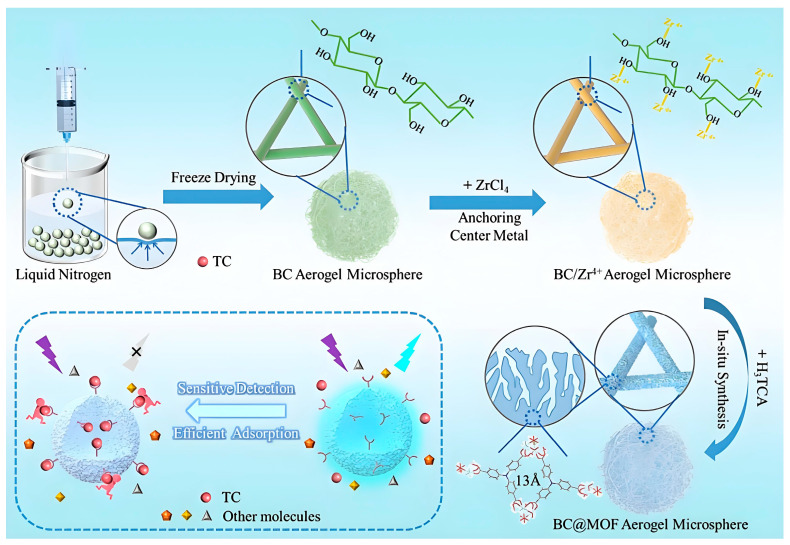
In situ growth of fluorescent Zr-MOF and construction of multifunctional material [10].

To enhance the performance of MOF-based multifunctional materials, some researchers have combined MOFs with other porous materials. For example, Wu and colleagues [10] grew Zr-MOF in situ on bacterial nanocellulose (BC), producing the composite BMAT_3_H_5_ for the simultaneous detection and adsorption of TC (Figure 3). The BC contributed to the stability of the MOF, and the molecular mass transfer efficiency was improved by the aerogel microspheres. The fluorescence detection of TC yielded a low LOD of 28 ± 0.012 nM and an adsorption capacity of 317.6 mg/g.

**Figure 4:**
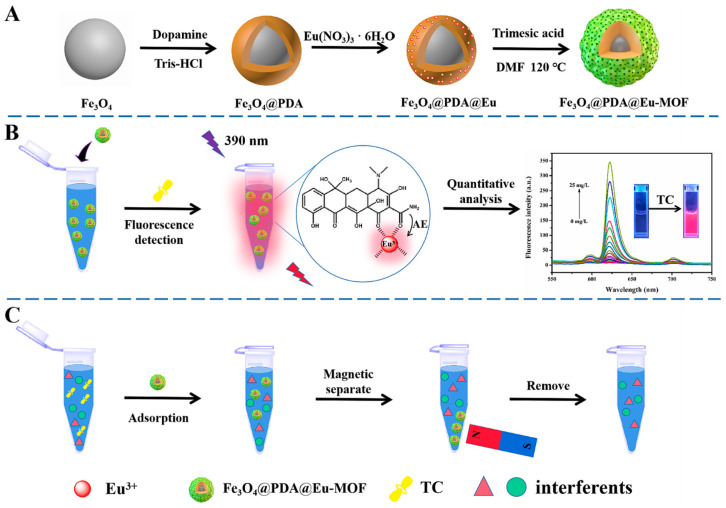
(**A**) Schematic illustration of preparation process for Fe_3_O_4_@PDA@Eu-MOF. (**B**,**C**) Detection and isolation of TC [11].

Pu and coworkers [11] prepared a magnetic MOF material, Fe_3_O_4_@PDA@Eu-MOF, using a layer-by-layer self-assembly method, as shown in Figure 4A. In this structure, Eu^3+^ coordinated with TC as a bidentate ligand, and the “antenna effect” (AE) sensitized Eu^3+^ to emit red light, making Fe_3_O_4_@PDA@Eu-MOF an effective sensor for TC detection with an LOD of 2 μg/L and excellent selectivity. Due to its large specific surface area, Fe_3_O_4_@PDA@Eu-MOF demonstrated a high adsorption capacity for TC (144.9 mg/g). Due to the β-diketone structure of TC, coordination between TCs and Eu^3+^ was generated, and π-π stacking was generated, which contributed to the adsorption. The core of Fe_3_O_4_ facilitated the magnetic separation of the catalyst after TC adsorption, and the material was successfully applied for TC detection and removal in milk and honey, demonstrating its broad application potential.

Other studies have also synthesized MOF-based materials capable of simultaneously detecting and removing antibiotics. For example, Zn-MOF with 2-(4-carboxyphenyl)-1H-benzo[d]imidazole-5-carboxylic acid (CBC) as a ligand was used for fluorescence detection and adsorption of OTC [51]. Yang’s work [52] utilized postsynthetic modification (PSM) to expand the functional group range of ZIF-8, creating a core–shell p-type semiconductor@MOF (Eu-CuBi_2_O_4_@ZIF-8) via in situ growth and postsynthetic metal exchange. This catalyst showed a low LOD and fast response during TC fluorescence detection and exhibited good adsorption performance. Additionally, the material’s color changed from dark to red upon UV irradiation in the presence of TC, facilitating visual detection. Wang et al. [53] also developed Europium-based MOF materials for simultaneous fluorescence detection and removal of TC, investigating mechanisms such as π-π interactions, electrostatic attraction, and hydrogen bonding. Researchers have also studied Eu-doped Zr-MOFs for TC fluorescence detection [54] and Cd-MOFs for simultaneous fluorescence detection and adsorption of chloramphenicol (CHL) [55].

The porosity and tunability of MOFs have significantly contributed to their application in the treatment of antibiotics. Other materials fall far short of MOFs, e.g., the crystal zeolite with a porous structure is inferior in terms of its adsorption efficiency and detection of antibiotics [77]. Despite these advances, much of the research has focused on tetracyclines (TCs), and there is limited investigation into the simultaneous detection and removal of other antibiotics, such as penicillin, cephalosporins, aminoglycosides, and macrolides. This is likely due to the unique characteristics of TCs, which are easier to detect and adsorb.

#### 2.1.2. Treatment of Other Organic Contaminants

**Figure 5:**
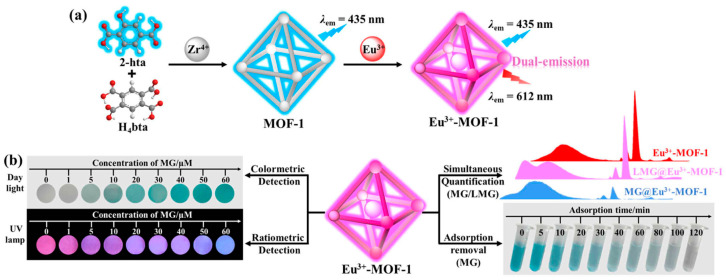
(**a**) The synthesis of Eu^3+^-MOF-1 and (**b**) the multifunctionalities of Eu^3+^-MOF-1 for the colorimetric and ratiometric detection of MG, the simultaneous quantification of MG and LMG, and adsorption-based removal of MG [15].

Malachite green (MG) is a dye commonly used in aquaculture and the fishery industry. However, MG can be reduced to a more toxic compound, leuco-malachite green (LMG), in organisms, posing significant health risks to humans and other species [78,79]. As shown in Figure 5, Bao et al. [15] developed a Eu^3+^-MOF material by modifying the UiO-66 precursor with red-emissive Eu^3+^ ions and a blue-emissive ligand through pre- and post-functionalization methods. The Eu^3+^-MOF demonstrated excellent selectivity and sensitivity for detecting MG and LMG, with limits of detection (LODs) of 34.20 nM and 1.98 nM, respectively, in fluorescence-based assays. Additionally, a paper-based sensor incorporating the Eu^3+^-MOF was developed, which, when combined with a smartphone, allowed for portable and efficient detection. Due to its high surface area and interactions such as π–π stacking, coordination bonding, and electrostatic interactions, the Eu^3+^-MOF exhibited excellent adsorption capacity for MG. These materials show great promise for applications in water treatment for food safety and environmental protection. Most dyes are organic compounds with different molecular structures; therefore, it is much easier for MOFs to interact with dye molecules by generating more various chemical bonds compared to other materials, such as activated carbon and clay [80]. However, MOF materials are not advantageous in terms of cost and stability.

2,4,6-Trinitrophenyl phenol (TNP) is a harmful environmental contaminant commonly used in industrial and military explosives [81]. Its toxicity and water solubility pose significant risks to human health. Wang and Yuan [56] developed a dual-functional MOF material made of Zr-Sti, capable of both detecting and removing TNP from aqueous solutions. Zheng and colleagues [57] also synthesized Cd-based MOFs for luminescence-based detection and removal of TNP. The Cd-MOF demonstrated fast, sensitive, and recyclable detection with an LOD of 0.38 μM. To enhance the adsorption capacity, the Cd-MOF was integrated with macroporous melamine foam (MF), resulting in the composite material Cd-MOF@MF, which exhibited an impressive adsorption capacity of 456.16 mg/g for TNP. This material was successfully applied to treat real samples, highlighting its potential for wastewater treatment. In the area of detection or degradation of phenols, many technics with high efficiency and low cost have been developed [82,83]; however, MOFs have unique advantages as multifunctional materials for phenol treatment.

**Figure 6:**
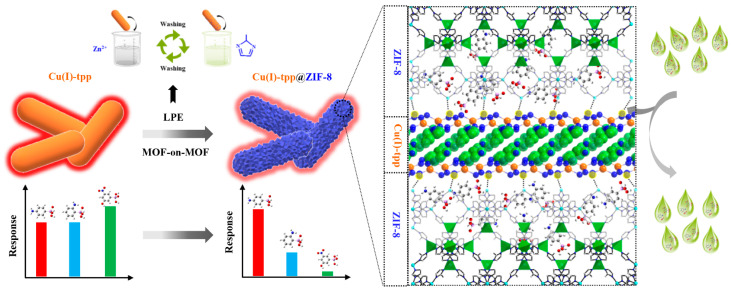
Schematic of Cu^+^-tpp@ZIF-8 fabrication process [58].

*P*-arsanilic acid (*p*-ASA), commonly used as a feed additive, has raised concerns due to its toxicity to aquatic ecosystems and its potential to affect human health. Yang et al. [58] synthesized a novel nitrogen-rich 2D MOF material, Cu^+^-tpp, which was then combined with ZIF-8 to form a heterostructure, Cu^+^-tpp@ZIF-8, using a liquid-phase epitaxy method, as depicted in Figure 6. This strategy overcame lattice mismatch issues due to the abundant nitrogen-rich sites. The Cu^+^-tpp@ZIF-8 material demonstrated excellent performance in the simultaneous fluorescence detection and adsorption of *p*-ASA, achieving an LOD of 0.4 µg/L and a high adsorption capacity of 303.0 mg/g. The excellent adsorption capacity should result from the synergistic effect of coordination interactions, hydrogen bonding, and π-π interactions.

**Figure 7:**
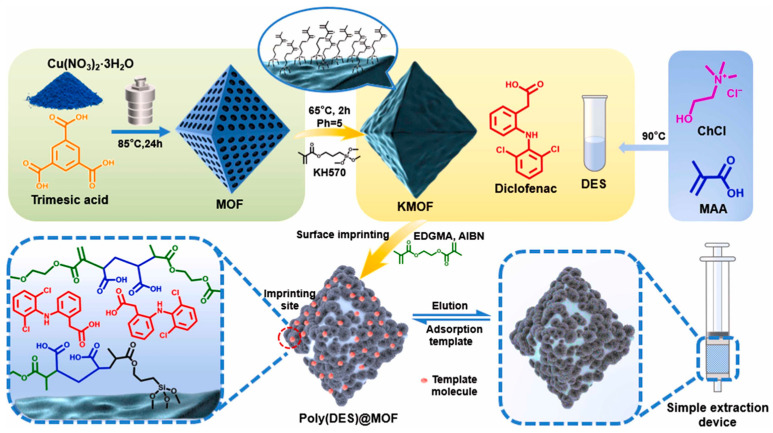
The synthesis process of the DES, MOF, and Poly(DES)@MOF [59].

Diclofenac, a commonly utilized non-steroidal anti-inflammatory drug (NSAID), may exert detrimental long-term impacts on both the environment and human health when released into the ecosystem [84,85]. Li and Du [59] developed a MOF-based material, poly(DES)@MOF, using surface imprinting. In this material, MOF-199, diclofenac, and a deep eutectic solvent (DES) act as the support, template, and functional monomer, respectively (Figure 7). The adsorption capacity for diclofenac, determined using UV spectrophotometry, is 19.39 mg/g, with an excellent recovery rate. This work broadens the application of MOF-based materials for pollutant detection and removal in wastewater.

Pesticides, widely used in agriculture to control diseases, weeds, and pests, have become a major environmental concern due to their persistence and potential toxicity. Every year, approximately 1–2.5 million tons of pesticides are used globally, and many end up in the environment, threatening ecosystems and human health [61]. Yang and colleagues [37] developed two MOF materials, [Zn(tpt)_2_·2H_2_O]_n_(Zn_1_) and [Zn_2_(tpt)_2_(bdc)]_n_(Zn_2_), for the simultaneous detection and removal of the pesticide thiophanate-methyl (TM) from water. After modification, Zn_2_ was combined with sodium alginate (SA) to form ZIF-8-on-Zn_2_@SA, which was applied for fluorescence detection of TM in vegetables and fruits, achieving an LOD of 0.14 μM. ZIF-8-on-Zn_2_@SA also exhibited an excellent adsorption capacity (161.8 mg/g) for carbendazim, a metabolite of TM. In another study, Yang’s group [37] synthesized {[Cd(tbia)⋅H_2_O]⋅2H_2_O}_n_-alginate-Ca^2+^-polyacrylic acid (kgd-M1@ACPs) for naked-eye detection with an LOD of 0.09 µM and an adsorption capacity of 83.3 mg/g for the pesticide 2,6-dichloro-4-nitroaniline. Yang et al. [61] also developed Zn2@ZIF-8@SA for the simultaneous fluorescence detection and removal of pesticides. This material exhibited low LODs of 0.08 µM for quinclorac (QNC), 0.09 µM for 2,6-dichloro-4-nitroaniline (DCN), and 0.37 µM for thiabendazole (TBZ). The adsorption capacity for QNC was 142.1 mg/g. Furthermore, a sensor test box was created for visual detection of pesticide residues on farm products. Similar to the antibiotics, most pesticides are organic molecules, which will facilitate treatment by MOFs. The tenability of MOFs including the pore size and the functional groups on the framework will greatly contribute to the application of MOF materials in monitoring and eliminating pesticides.

### 2.2. Inorganics

#### 2.2.1. Heavy Metal Ion Treatment

The presence of heavy metals in wastewater, such as copper, zinc, nickel, mercury, lead, cadmium, and chromium, poses significant risks to both ecosystems and human health. These metals are non-biodegradable and can accumulate in organisms, leading to serious health problems [86,87,88]. Therefore, the treatment of heavy metal ions in wastewater has become a critical area of research.

Mercury, a neurotoxic trace metal, poses significant environmental and human health risks due to its accumulation and biomagnification in the food chain [89]. It can cause reproductive issues, neurological disorders, and developmental disabilities. Consequently, extensive efforts have been dedicated to address mercury pollution. You et al. [62] synthesized a hierarchical MOF-on-MOF hybrid by embedding gold nanoclusters (AuNCs) into ZIF-8, which was then applied for the simultaneous detection and adsorption of Hg^2+^. The AuNCs, confined within the ZIF-8 layer, triggered aggregation-induced emission, enhancing fluorescence upon Hg^2+^ interaction. The hybrid material, UiO-66-NH_2_@AuNCs/ZIF-8, demonstrated an LOD of 0.42 ppb and an adsorption capacity of 129.9 mg/g, which were comparable to that of most reported adsorbents for Hg^2+^. It was found that the complexation between Hg^2+^ and N-containing groups in ZIF-8 or UiO-66-NH_2_ and the Hg^2+^-Au^+^ interaction contributed to the super adsorption capability. This study provided an effective MOF-based material for both the detection and removal of mercury. Yang and colleagues [63] developed a MOF-based material containing amino groups for the simultaneous detection and adsorption of Hg^2+^. The charge transfer emission of the material was effectively quenched in the presence of Hg^2+^, with a low LOD of 0.87 μM. The material also exhibited excellent adsorption, removing 97% of Hg^2+^ within 2 h from a 0.1 M solution.

A more convenient naked-eye detector for Hg^2+^ was developed by Safaei et al. [64] using thiazolo[5,4-d]thiazole, a ligand with a strong affinity for mercury. This ligand was incorporated into Zn_2_(BDC)_2_(TzTz)_2_, which exhibited a fluorescence color change from light cream to fluorescent yellow within 3 min upon Hg^2+^ adsorption. The material showed an impressive adsorption capacity of 1428 mg/g for Hg^2+^, with excellent selectivity and stability. Similarly, El-Sewify et al. [65] designed thioketone-functionalized Al-MOFs (TAM) for colorimetric detection and adsorption of Hg^2+^, where Hg^2+^ binding with TAM caused a color shift from yellow to green. The LOD was calculated to be 0.8 ppb, and the TAM showed a good adsorption capacity of 1110 mg/g for Hg^2+^.

Hexavalent chromium (Cr_2_O_7_^2−^) is widely used in industries such as printing, dyeing, steel manufacturing, and more. However, even low concentrations of Cr_2_O_7_^2−^ are carcinogenic and teratogenic, raising concerns about its environmental pollution [90,91]. Wang et al. [56] developed a Zr-based MOF, Zr-Sti, to simultaneously detect and remove Cr_2_O_7_^2−^. The material exhibited a fluorescence resonance energy transfer mechanism, achieving an LOD of 0.73 μM (159 ppb) for Cr_2_O_7_^2−^ and an adsorption capacity of 43.38 mg/g resulting from the hydrogen bonds. Zr-Sti also showed effectiveness in removing 2,4,6-trinitrophenyl phenol.

**Figure 8:**
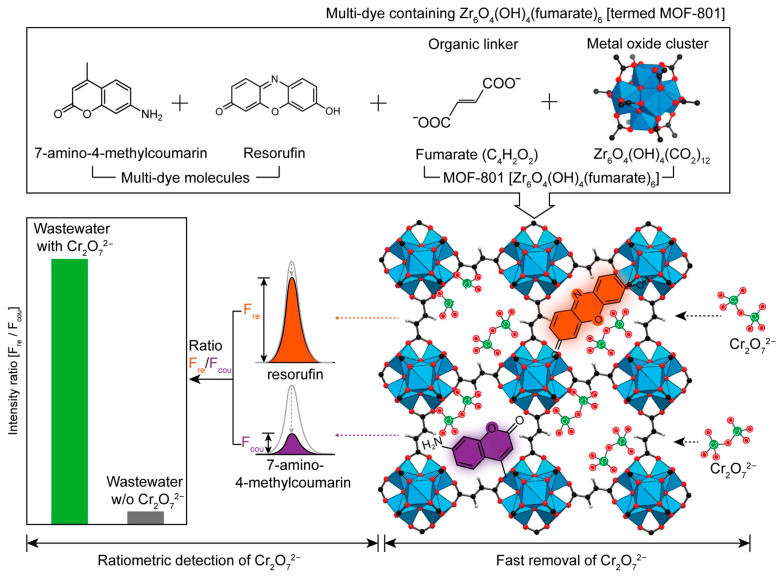
Schematic of treatment of Cr_2_O_7_^2−^ using Dyes⊂MOF-801 [66].

Yoo et al. [66] encapsulated coumarin and resorufin dye molecules into MOF-801, forming Dyes⊂MOF-801, which was used for the ratiometric fluorescence detection and removal of Cr_2_O_7_^2−^, as shown in Figure 8. The dual-emission property of this material allowed for the detection of Cr_2_O_7_^2−^ at 0.03 mM, even in the presence of 260 times higher concentrations of interfering ions. Dyes⊂MOF-801 also showed a high adsorption capacity of 83 mg/g for Cr_2_O_7_^2−^ within 3 min.

Kataria’s group [68] synthesized a highly stable Zn-based MOF (PUC-5) using 1-(3-aminopropyl)imidazole and trimesic acid. PUC-5 was used to detect and remove Fe^2+^, Pb^2+^, and V^5+^ from water. The interaction between these metal contaminants and the -C=O groups on PUC-5 caused a significant hyperchromic shift in the absorption peaks. The LODs for Pb^2+^, Fe^2+^, and V^5+^ were 0.113, 0.129, and 0.246 µM, respectively. PUC-5 showed excellent performance in real water samples, including seawater, groundwater, and tap water. The material also exhibited a high adsorption capacity of 208.7 mg/g for Fe^2+^, 192.6 mg/g for Pb^2+^, and 203.6 mg/g for V^5+^.

Wang’s group [69] synthesized amino-functionalized MIL-101(Fe) using a simple one-step method. In this system, the organic linkers with amino groups were only needed for fluorescence emission. Because of the chelation between the metal ions and the amine groups, the material exhibited excellent detection capabilities for Fe^3+^, Cu^2+^, and Pb^2+^ ions, with LODs of 1.8, 1.6, and 5.2 μM, respectively, and adsorption capacities of 3.5, 0.9, and 1.1 mM/g. The adsorption might result from the coordination between the C=O unit and metal ions. Similar performance was observed for MIL-53-NH_2_(Al), MIL-101-NH_2_(Cr), MOF-5-NH_2_(Zn), and UiO-66-NH_2_(Zr).

While certain heavy metal ions, such as Fe^3+^, Cu^2+^, and Zn^2+^, are essential for the human body, imbalances (either excess or deficiency) can lead to disease [92]. Therefore, it is important to develop techniques for the detection and removal of these ions. Wen et al. [67] synthesized a 2D layered MOF (HSB-W15) combining 5-aminoisophthalic acid and 1,2-bis(4′-pyridylmethylamino)-ethane ligands. Then, HSB-W15-NS was observed by means of instant in situ exfoliation. Due to the specific structure of the ultrathin nanosheets and the abundant active sites on the surface, HSB-W15-NS was proven to be an excellent fluorescent sensor for detecting Fe^3+^ with an LOD of 0.837μM. In addition, Fe^3+^ could be selectively captured with a high adsorption capacity (250.81 mg/g) within 5 min. It was found that the free carbonyl groups, amino, and pyridyln contributed to the high adsorption capacity.

Other heavy metal ions, such as As^5+^ and Cd^2+^, have also been studied for simultaneous detection and adsorption. Cd^2+^ was treated using Fe_3_O_4_/MOF/L-cysteine for fluorescent detection with an LOD of 0.94 ng/mL and an adsorption capacity of 248.24 mg/g [70]. As^5+^ was detected and adsorbed by an amino-functionalized Fe-based MOF, with an LOD of 4.2 ppb and a maximum adsorption capacity of 125 mg/g [71].

As we all know, zeolites including natural, modified, and synthetic zeolites are also ideal materials for the treatment of heavy metal ion pollution [93]. The adsorption of heavy metal ions by zeolite through sorption and ion exchange depends on the charge density and hydrated ion diameters [94]. In addition, in terms of cost, zeolites have a big advantage. However, for the detection of heavy metal ions, MOFs have an absolute advantage.

#### 2.2.2. Inorganic Anion Treatment

Phosphorus is an essential nutrient for all life forms, playing a key role in biochemical processes. However, phosphorus pollution, often leading to eutrophication and algal blooms in aquatic ecosystems, threatens the environment and human health [95,96]. Therefore, addressing phosphorus pollution is critical. Wang’s group [72] synthesized a luminescent, rod-like terbium-based MOF (Tb-BTC) via a hydrothermal method for phosphorus detection and adsorption. Tb-BTC exhibited a fluorescence quenching effect, achieving an LOD of 2.97 μM, and a maximum adsorption capacity of 222.2 mg/g resulting from the electrostatic attraction. To improve operability and recoverability, Tb-BTC was integrated onto polyacrylonitrile nanofibers to form a nanofibrous membrane for phosphorus treatment. However, the performance decreased slightly compared to pure Tb-BTC.

**Figure 9:**
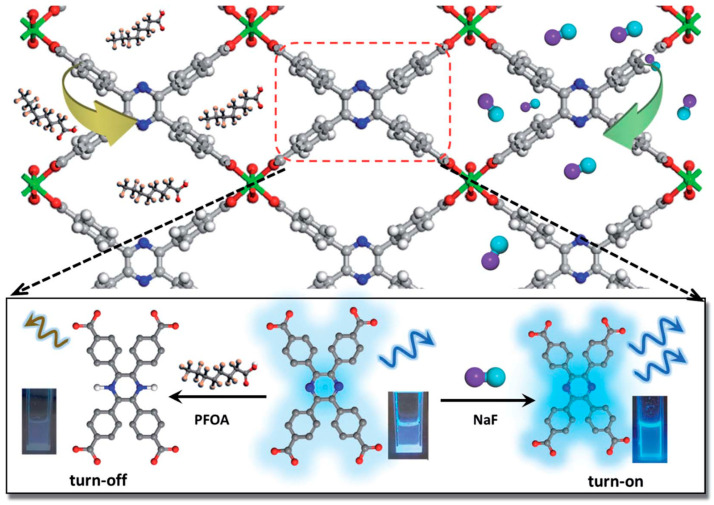
The structure of In(tcpp) and its luminescence switchable turn-off and turn-on response [73].

Fluoride (F^−^) plays a crucial role in human health by contributing to fluorapatite formation in bones and teeth [97]. However, fluoride intake can be toxic when its concentration is higher than 0.05 mg/Kg/day. Li et al. [73] synthesized a luminescent MOF made of In(tcpp) with the chromophore ligand 2,3,5,6-tetrakis(4-carboxyphenyl)pyrazine (H_4_tcpp). As depicted in Figure 9, in(tcpp) served as a “switchable” sensor, exhibiting turn-on and turn-off photoluminescence signals when complexed with F^−^ and perfluorooctanoic acid (PFOA), with LODs of 1.3 mg/L for F^−^ and 19 mg/L for PFOA. In(tcpp) also demonstrated excellent adsorption capacities of 36.7 mg/g for F^−^ and 980.0 mg/g for PFOA. The bridging -OH^−^ between In(tcpp) and F^−^, the N in the component, and the acid–base interaction between the PFOA and In(tcpp) contributed to the excellent adsorption.

Radioactive iodine isotopes (^129^I and ^131^I), produced during nuclear energy generation, are highly toxic to the environment and human health [98]. Therefore, developing treatment methods for iodine pollution is very important for protecting the environment and human health. Zhang et al. [74] developed a multifunctional material by decorating silver ions (Ag^+^) onto nano-MOF UiO-66-(COOH)_2_ for the simultaneous detection and removal of iodide (I^−^) from aqueous solutions. By connecting with the carboxylate groups, Ag^+^ was incorporated onto UiO-66-(COOH)_2_, enhancing the fluorescence of the MOF. However, in the solution containing I^−^, Ag^+^ on UiO-66-(COOH)_2_ reacted with I^−^, producing AgI. Therefore, the fluorescent signal decreased and I^−^ transformed into precipitate AgI simultaneously. The LOD was calculated to be 0.58 ppm and the adsorption capacity was 235.5 mg/g.

The simultaneous detection and removal of contaminants is an effective method for wastewater treatment. However, several challenges need to be addressed. (1) After adsorbing contaminants such as antibiotics, MOFs need to be regenerated (i.e., desorbed) for reuse. This process often requires large amounts of solvents or water, increasing both costs and the need for MOFs to maintain high stability. (2) MOFs can be sensitive to factors such as pH, temperature, and solvent exposure, which can affect their stability and durability. Therefore, enhancing the stability of MOFs is essential. (3) Scaling up the production of MOFs and integrating them into industrial-scale processes presents significant challenges.

## 3. Simultaneous Detection and Degradation of Contaminants

MOF materials are known for their porous structures, which enable the effective adsorption for contaminants. This property makes them suitable for a wide range of applications, such as catalysis, water purification, and gas separation [20,99]. When designing multifunctional MOF-based materials, the ability to adsorb contaminants is often prioritized [21,36]. Most studies on multifunctional MOF materials focus on this adsorption capacity, with additional functions such as photocatalysis, Fenton-like reactions, and other catalytic activities being integrated [100,101,102]. As a result, many MOF materials combine both degradation and sensing abilities, as shown in Table 2. However, challenges remain in optimizing the components and minimizing interference, highlighting the need for innovative materials to enhance catalyst performance [36].

### 3.1. Antibiotics

**Figure 10:**
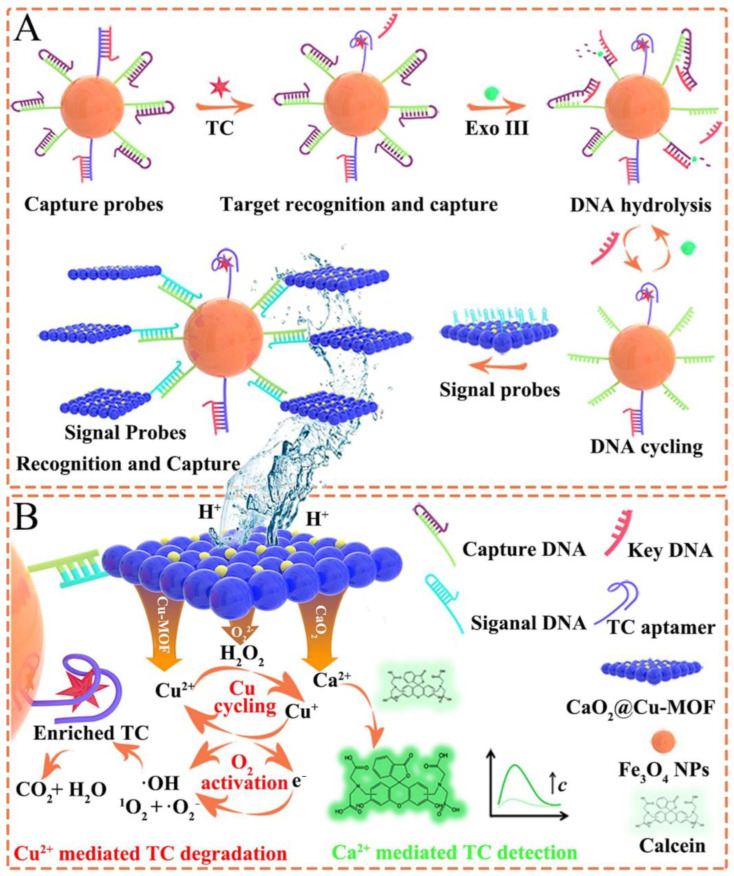
Illustration of (**A**) the Exo III-mediated DNA recycling reaction; (**B**) acid-initiated bimetallic ion-mediated TC fluorescence detection and Fenton-like degradation. [21].

Due to their widespread use and the resulting environmental pollution, antibiotics are commonly targeted as contaminants [103,104]. In a study by Li et al. [21], CaO_2_-loaded Cu-MOF nanosheets (CaO_2_@Cu-MOF NSs) were developed for the simultaneous detection and degradation of tetracycline (TC). As shown in Figure 10, TC in solution was adsorbed onto CDNA@Fe_3_O_4_ NPs. The signal probes were dissociated by acid, releasing Ca^2+^, Cu^2+^, and H_2_O_2_. The fluorescence of calcein was activated by Ca^2+^, enabling TC detection. Simultaneously, Cu^2+^ and H_2_O_2_ facilitated Cu^2+^/Cu^+^ cycle-mediated Fenton-like oxidation to degrade TC. The system achieved a low LOD of 11.8 fg/mL, with 95% of the 40 mg/L TC degraded within 60 min. Their study shows great promise for practical applications.

Some MOF materials also exhibit photocatalytic activity; therefore, some researchers have constructed MOF-based heterojunctions for photodegradating contaminants. For example, Han’s group [36] combined In_2_S_3_ with Zr-MOF (PCN-224) to create a Z-scheme heterojunction (In_2_S_3_@PCN-224) for TC detection and photodegradation. When TC interacted with the In_2_S_3_@PCN-224 sensor, the fluorescence intensity decreased due to strong interactions between TC and the porphyrin ligands. The LOD for TC was 55 nM. Due to the successful construction of the heterojunction, a 25 mg/L TC solution was treated with a degradation rate of approximately 80%, owing to both adsorption and photodegradation effects. This work extends the concept of the “integration of diagnosis and treatment” in environmental management.

Our group have never tried to detect and degrade antibiotics simultaneously using modified zeolites [4]. Compared to zeolite materials, the functional variety of MOFs is an absolute advantage, because the modification of functional groups or nanoparticles is so limited. However, in terms of cost and stability, zeolite-based materials are much better; therefore, the improvement in stability and the reduction in cost are very important factors limiting the application of MOFs in at a large scale.

### 3.2. Phenolic Compounds

Phenolic compounds, such as phenols, chlorophenols, and derivatives, are commonly found in wastewater from industries like phenolic resin production, plastic manufacturing, petroleum refining, dye and textile industries, and agrochemical production [105,106]. These compounds are highly toxic and persistent in the environment, necessitating effective treatment before wastewater is released.

For decades, the integration of chemo- and enzyme catalysis in wastewater treatment has shown significant promise but also presents certain challenges [100,101]. Yin and coworkers [100] immobilized laccase (Lac) onto Cu_2_O@MOF through covalent linkage to form Cu_2_O@NMOF-Lac, which was used for the simultaneous detection and degradation of 2,4-dichlorophenol (2,4-DCP). In the UV colorimetric detection system, Cu_2_O@NMOF-Lac catalyzed the reaction between 2,4-DCP and 4-aminoantipyrine (4-AAP), producing a red dye. The LOD was 0.29 µM. In degradation experiments, hydroxyl radicals from H_2_O_2_ decomposition were found to be the primary active species, achieving an 82.35% degradation rate of 2,4-DCP (20 mg/L) in 2 h. Their work provides a platform combining enzymes and Cu_2_O@MOF for treating 2,4-DCP, although the authors did not investigate the adsorption capacity of the catalyst for 2,4-DCP.

**Figure 11:**
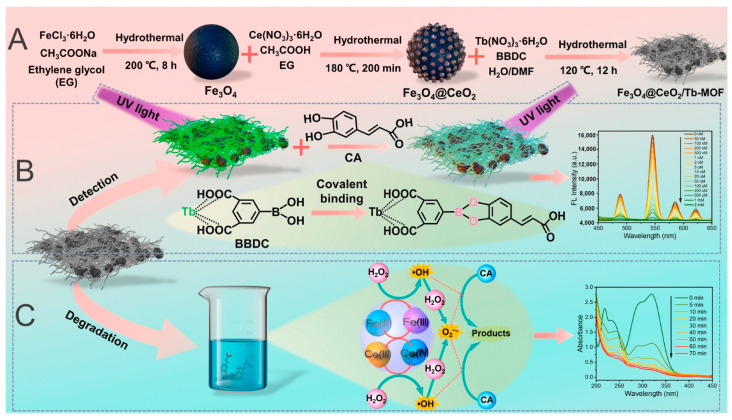
(**A**) Preparation of Fe_3_O_4_@CeO_2_/Tb-MOF using a multi-step hydrothermal method. (**B**) Sensing principle of Fe_3_O_4_@CeO_2_/Tb-MOF for CA by boric acid as a recognition group. (**C**) Catalytic mechanism of Fe_3_O_4_@CeO_2_/Tb-MOF nanozyme for the degradation of CA by producing reactive oxygen species [100].

Caffeic acid (CA), a natural polyphenol, is a common contaminant in wastewater from wine and olive oil production. CA concentrations often exceed the allowable limit of 0.5 mg/L [107,108]. Although CA has health benefits, its high concentration can harm bacteria, plants, and aquatic organisms. Therefore, CA pollution warrants attention. In the work by Wang and Chen [100], a magnetic luminescent nanozyme (Fe_3_O_4_@CeO_2_/Tb-MOF) was developed for the simultaneous detection and degradation of CA, as shown in Figure 11. In the presence of CA, the fluorescence of Fe_3_O_4_@CeO_2_/Tb-MOF decreased significantly due to energy competition absorption, nucleophilic reactions, and photo-induced electron transfer. The system achieved a low LOD of 18.9 nM, with a wide linear detection range from 50 nM to 500 μM. The modification of Fe_3_O_4_ and CeO_2_ imparted peroxidase activity to Fe_3_O_4_@CeO_2_/Tb-MOF, enabling CA degradation via free radicals generated from H_2_O_2_ catalysis.

### 3.3. Other Contaminants

Ibuprofen (IBP), a widely used pharmaceutical, has been detected in numerous water bodies, raising concerns about its potential environmental impact [109,110]. To address this issue, various strategies have been developed. Garg et al. [111] synthesized a hybrid material by combining ZnO and NH_2_-MIL-125(Ti), which was used for the simultaneous detection and degradation of ibuprofen. In the presence of IBP, the absorbance of the hybrid material greatly increased during fluorescence measurements due to their interaction. The LOD reached 0.15 μM within a linear range of 0 to 0.75 μM. In addition, the hybrid material demonstrated excellent photocatalytic activity for degrading IBP in real water samples, achieving a degradation efficiency of 88.9% at pH 5.7. It showed great potential as a multifunctional tool for detecting and treating IBP in wastewater.

The release of human and livestock waste, pharmaceuticals, and agricultural chemicals contributes to the production of estrogens, including 17*β*-estradiol (E_2_), which is commonly found in aquatic environments [112]. Even at concentrations as low as 1 ng/L, E_2_ can disrupt the endocrine systems of aquatic organisms [113]. Wang and Chen [102] developed an artificial nanozyme, Tb-OBBA-Hemin, for the simultaneous detection and degradation of E_2_ and its derivatives. This nanozyme consists of a luminescent Tb^3+^ ion, a light-harvesting ligand, and a catalytic coenzyme (hemin). In fluorescent detection, the LOD for E_2_ was 50 pM. The degradation rate of E_2_ reached 88% within 60 min, facilitated by the active ·OH and high-valent iron-oxo species. Tb-OBBA-Hemin holds promise as a substitute for the traditional combination of natural enzymes and chromogenic substrates in environmental applications.

**Table 2:** Summary of MOFs for simultaneous detection and degradation of contaminants.

Catalyst	Contaminant	Detection		Adsorption Capacity	Degradation		Year	Ref.
		Method	LOD		Method	Rate		
CaO_2_@Cu-MOF	TC	Fluorescent	11.8 fg/mL	-	Fenton-like	95% per 60 min	2024	[21]
In_2_S_3_@PCN-224	TC	Fluorescent	55 nM	60%	Photocatalysis	20%	2023	[36]
Cu_2_O@NMOF-Lac	2,4-dichlorophenol	UV colorimetric	0.29 µM	-	Peroxidase-like	82%	2023	[100]
Fe_3_O_4_@CeO_2_/Tb-MOF	Caffeic acid	Fluorescent	18.9 nM	-	Peroxidase-like	95%	2024	[101]
ZnO/NH_2_-MIL-125 (Ti)	Ibuprofen	Fluorescent	0.15 μM	~25%	Photocatalytic	88.9%	2024	[111]
Tb-OBBA-Hemin	17β-estradiol	Fluorescent	50 pM	-	Peroxidase-like	88%	2020	[102]

The simultaneous detection and degradation of contaminants is an ideal method for wastewater treatment. Unlike simultaneous detection and removal, which requires desorption of contaminants (particularly organic ones), the combination of detection and degradation (including adsorption and catalytic reactions) is more complex due to the need to integrate multiple functions into a single system. This complexity may explain why there are fewer studies on simultaneous detection and degradation compared to those focusing on detection and adsorption. Future research should focus on improving the stability and reactivity of MOFs, developing scalable and cost-effective synthesis methods, and understanding the environmental impacts of MOFs, along with sustainable disposal strategies.

## 4. One Stone, Two Birds

Wastewater contains a variety of contaminants, both organic and inorganic, which differ in chemical properties, solubility, and toxicity [114,115]. This diversity makes it challenging to remove all contaminants using a single material. Therefore, effective treatment requires the development of materials with multiple functionalities. To address this, researchers are focusing on creating multifunctional materials capable of targeting multiple contaminants simultaneously or sequentially, akin to the saying “killing two birds with one stone”.

### 4.1. Different Functions for Different Contaminants

The development of MOF-based multifunctional materials tailored for different contaminants offers significant potential for addressing environmental challenges [40,116,117]. With their highly porous structures, large surface areas, and tunable chemical properties, MOFs are excellent candidates for a variety of applications, including adsorption, separation, catalysis, and sensing. When treating wastewater with diverse contaminants, various MOF functions are often employed, as shown in Table 3.

Kataria et al. [116] fabricated a novel Zn-MOF@MCHS composite using an in situ method, combining mesoporous carbon hollow spheres (MCHS) and MOF-based fluorescent nanocomposites. This material was used for detecting 2,4,6-trinitrophenol (TNP) and Cu^2+^ and for the adsorption removal of Cu^2+^. The high surface area and unique structure of Zn-MOF@MCHS enhanced energy or charge transfer, resulting in an LOD of 0.301 µM for TNP and 0.368 µM for Cu^2+^. The adsorption capacity for Cu^2+^ reached 523.56 mg/g, with a removal efficiency of 99%.

**Figure 12:**
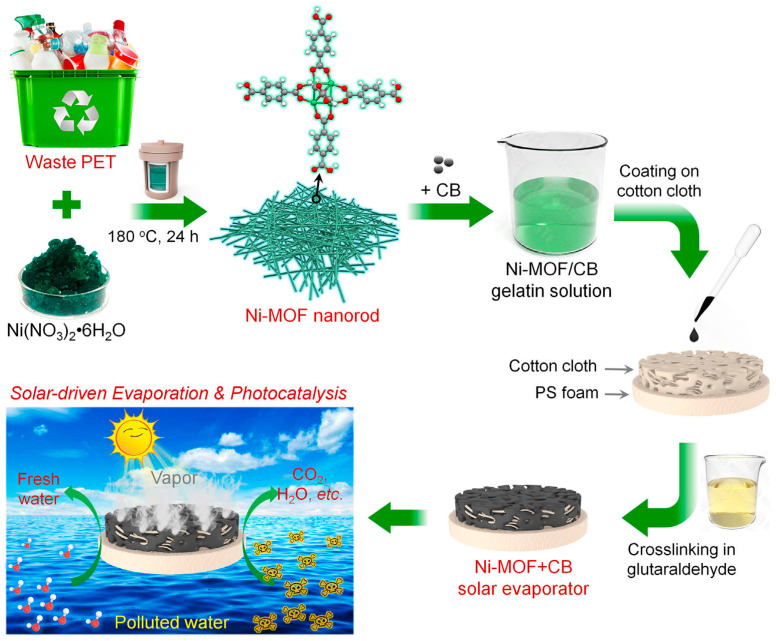
Scheme of fabricating Ni-MOF/carbon black evaporator for simultaneous water evaporation and photodegradation of tetracycline [40].

When obtaining freshwater by means of solar evaporation with wastewater, treatment of the accumulated contaminants in the bulk water is an important issue. Fan et al. [40] developed Ni-MOF nanorods from waste poly(ethylene terephthalate) (PET) for the simultaneous solar evaporation and photodegradation of antibiotics, as shown in Figure 12. These nanorods demonstrated excellent light absorption, high photothermal conversion, and a low vaporization enthalpy, achieving an evaporation rate of 2.25 kg/m^2^/h. Additionally, in peroxymonosulfate activation, the system achieved a 91% removal efficiency for tetracycline, driven by local heat and Ni-sites in the Ni-MOF. This approach offers a sustainable method for freshwater production and organic pollutant degradation.

The simultaneous detection of organic pollutants and removal of inorganic pollutants is a common focus in MOF-based multifunctional material research. For instance, Shahid’s group [118] developed a Co-MOF@CNT composite which enabled the simultaneous detection of Cr^6+^ and the removal of organic dyes. The system showed an impressive LOD of 0.125 μM for Cr^6+^ with excellent selectivity. The adsorption capacities for methylene blue and methyl orange were 98% and 72%, respectively.

Eu^3+^-based cationic MOFs, such as the one developed by Li et al. [117], exhibit excellent sensing performance for antibiotics like nitrofurantoin and nitrofurazone, with low LODs of 1.33 μM and 2.80 μM, respectively. Additionally, Eu-CMOF demonstrated a high adsorption capacity of 1.1 g/g for MnO_4_^−^ due to its cationic framework and decentralized positive charges.

Another example includes TMU-57, a MOF material functionalized with thiophene-urea groups, designed for detecting nitroaromatic compounds (NACs) and adsorbing Hg^2+^ [119]. Through hydrogen bonding between the hydroxyl group of TNP and the urea functionality of TMU-57, a low LOD of 2 ppb was achieved for TNP detection. This demonstrates the versatility of multifunctional MOFs in addressing multiple applications. Similarly, Wang’s group [120] also developed MOFL-TpBD for the simultaneous fluorescence detection of TNP and lead adsorption, further illustrating the versatility of MOF-based materials.

In the realm of biomimetic detection, Huang and colleagues [121] developed an MOF-based nanozyme (CA-Cu) with laccase- and catecholase-like activities. The active site, formed by the coordination between nitrogen and copper, enabled high-performance dopamine detection with an LOD of 2.23 μM and efficient degradation of chlorophenol and diphenol (80% and 50%, respectively, within 8 h). Their work highlights the potential of MOF-based nanozymes for multifunctional applications in contaminant detection and degradation.

MOFs can also serve as pH sensors. For instance, Wang and Li [122] developed a luminescent Zr-MOF (Zr-BBI) that exhibited a sensitive fluorescence response to pH changes between 4.6 and 7.12. Zr-BBI also demonstrated excellent performance in detecting Cr_2_O_7_^2−^ with a low LOD of 0.69 μM, and it could reduce Cr^6+^ to Cr^3+^ under visible light, enhancing its photocatalytic activity.

**Table 3:** One MOF with different functions for different contaminants.

Catalyst	Removal		Detection		Degradation		Other Function	Year	Ref.
	Contaminant	Adsorption Capacity and Removal Rate	Contaminant	Method and LOD	Contaminant	Method and Rate			
Zn-MOF@MCHS	Cu^2+^	523.56 mg/g, 99%	2,4,6-trinitrophenol (TNP), and Cu^2+^	Fluorescent, 0.301 and 0.368 µM	-	-	-	2025	[116]
Ni-MOF	-	-	-	-	TC	Peroxymonosulfate, 91%	Interfacial solar evaporation (2.25 kg/m^2^/h)	2023	[40]
Co-MOF@CNT	Methylene blue and Methyl orange	98% and 72%	Cr^6+^	Fluorescent, 0.125 μM	-	-	-	2022	[118]
TMU-57	Hg^2+^	570 mg/g	2,4,6-trinitrophenol	Fluorescent, 2 ppb	-	-	-	2022	[119]
MOFL-TpBD	Pb^2+^	21.74 mg/g	2,4,6-trinitrophenol	0.32 μg/L	-	-	-	2021	[120]
Eu-CMOF	MnO_4_^−^	1.1 g/g	Nitrofurantoin and Nitrofurazone	Fluorescent, 1.33 and 2.80 μM	-	-	-	2022	[117]
CA-Cu	-	-	Dopamine	UV colorimetric, 2.23 μM	chlorophenol and diphenol	Laccase-like, 80% and 50%	-	2022	[121]
Zr-BBI	-	-	-	-	Reducing Cr_2_O_7_^2−^ to Cr^3+^	Photocatalysis, k = 0.073 min^−1^	pH sensor (pH 4.6–7.12)	2021	[122]

In summary, the development of MOF-based multifunctional materials, tailored to address various contaminants, represents a promising direction for environmental remediation. By optimizing the design and properties of these materials, researchers are creating effective, reusable, and environmentally friendly solutions for wastewater treatment and pollutant detection. In addition, the tunability of MOF materials make them more idealized materials than biomass, activated carbon, and zeolite materials.

### 4.2. One Function for Different Contaminants

#### 4.2.1. Removal of Various Contaminants

MOFs are highly effective materials for contaminant adsorption in aquatic environments due to their extremely high surface areas, which provide abundant adsorption sites, and their tunable pore sizes, which allow for the selective targeting of different contaminants [3,16,20]. The diverse functionalities of MOFs further enhance their capacity to adsorb a wide range of pollutants. As a result, MOFs have been widely studied for removing various contaminants from water, as shown in Table 4.

Water contamination is a serious environmental issue, with diverse sources of pollution in wastewater [123,124]. Thus, developing efficient materials for wastewater treatment is essential. Pardo and Armentano [16] developed a novel, water-stable, multivariate (MTV) MOF with oxamide-based metalloligands. This MOF features hexagonal channels decorated with -CH_2_OH and -CH_2_CH_2_SCH_3_ groups, enabling it to effectively remove both organic contaminants (e.g., dyes) and inorganic contaminants (e.g., Pb^2+^, Tl^+^, and Hg^2+^). Additionally, MTV-MOF exhibited excellent reusability, marking the first report of an MOF material that can remove both organic and inorganic contaminants from water, thus demonstrating broad application potential for wastewater treatment.

Following the above study, many researchers have focused on the simultaneous removal of both organic and inorganic contaminants. For example, Bhat and colleagues [125] synthesized a Zr-based MOF for removing the organic contaminant methylene blue and the inorganic contaminants lead and cadmium ions from wastewater. Zr-MOF’s zeta potential of −7.7 mV at a neutral pH facilitated strong interactions with the adsorbates. Singh et al. [126] fabricated a CaFu MOF, which was used for the simultaneous adsorption of the pesticide imidacloprid and Cd^2+^ ions from an aqueous ecosystem.

Several researchers have also combined MOFs with other materials to enhance their ability to simultaneously remove both organic and inorganic contaminants. For example, Li and coworkers [127] modified carboxymethylated filter paper (CMFP) by means of layer-by-layer deposition of NH_2_-Cu-BDC, forming Cu-MOFs/CMFP. This composite material efficiently captured dyes and metal ions (Pb^2+^ and Cd^2+^), with a removal efficiency of nearly 90% when 30 layers of Cu-MOFs/CMFP were used. The study introduced a novel approach for designing MOF-based materials capable of removing both organic and inorganic contaminants simultaneously. Similarly, porous ZnO microspheres were combined with Zn-MOF-74 to create ZnO-NP@Zn-MOF-74, which exhibited excellent adsorption performance for Cu^2+^ and tetracycline (TC), achieving 106.27 mg/g for Cu and 137.17 mg/g for TC due to interactions like π-π stacking, surface complexation, electrostatic interactions, and ion exchange [17]. Composites of ZIF-8 MOFs and multi-walled carbon nanotubes (MWCNTs) were developed for removing phosphate and emerging organic contaminants (EOCs), such as acetaminophen (AAP) and triclosan (TCS) [128]. The composites exhibited a maximum adsorption capacity of 188.5 mg/g for phosphate, likely due to Zn-O-P interactions and hydrogen bonding. However, the presence of EOCs hindered phosphate adsorption.

**Figure 13:**
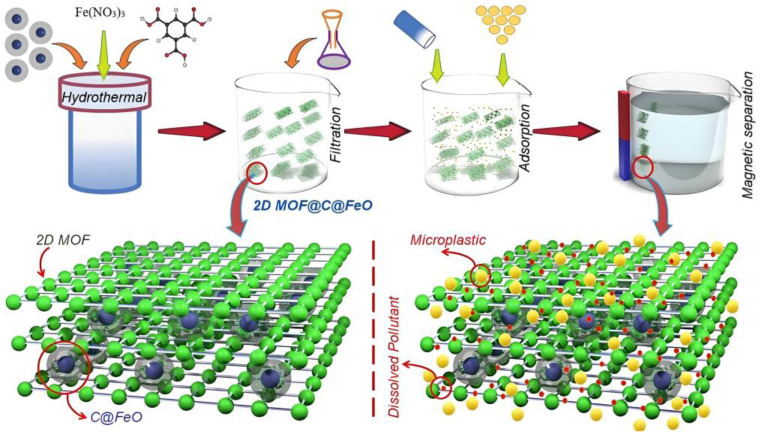
Illustration of preparation process of nanopillared MOF@C@FeO, adsorption process, magnetic separation, and potential microplastic removal pathway [129].

Some MOF-based materials have also been applied to remove different organic contaminants simultaneously. For instance, C@FeO nanopillars were combined with a 2D MOF to create 2D-MOF@C@FeO, which was used for the simultaneous removal of microplastics (MP) and dissolved contaminants like methylene blue (MB), as shown in Figure 13 [129]. 2D-MOF@C@FeO exhibited a high surface area, magnetic properties, and abundant active sites, achieving a 100% removal rate for MP alone and a 90% removal rate for both MP and MB after six adsorption cycles. Similarly, Rafiee et al. [30] developed a Zn-based MOF hybridized with a covalent organic framework (COF), forming MOF-5/COF(M5C), which was used for the simultaneous removal of rhodamine B (RB) and auramine O (AO) cationic dyes. The composite exhibited adsorption capacities of 17.95 and 16.18 mg/g for AO and RB dyes, respectively, due to hydrogen bonding, π–π stacking, electrostatic interactions, and Lewis acid–base interactions. Different antibiotics [9] and dyes [15] were also studied.

MOFs are also effective for removing various inorganic contaminants simultaneously. Yang and colleagues [130] synthesized thiol-functionalized defective Zr-MSA-DMSA by mixing mercaptosuccinic acid and 2,3-dimercaptosuccinic acid which was used to remove metal ions from wastewater. The maximum adsorption capacities for Pb^2+^, Hg^2+^, and Cd^2+^ were 715.2, 862.7, and 450.5 mg/g, respectively. The unsaturated adsorption sites and oxygen vacancies on Zr-MOF contributed to its ability to adsorb heavy metal ions. Further investigation revealed that electrostatic attraction, abundant defective sites, and coordination between oxygen and sulfur atoms played key roles in metal ion adsorption. In another study, Sridhar et al. [77] synthesized mixed-matrix composites of NH_2_-MIL-101-Fe and MOF-808-EDTA, which were applied for the removal of heavy metal ions from wastewater. The MOF-808-EDTA spheres exhibited the best performance, with adsorption capacities of 272.7, 151.29, and 125.9 mg/g for Hg^2+^, As^3+^, and Mn^2+^, respectively, at a pH of 5–5.5. Maspoch and colleagues [131] developed composite microbeads of a MOF (UiO-66 or UiO-66-(SH)_2_) and CeO_2_ via continuous-flow spray-drying. These composites were used to simultaneously remove multiple metal ions, including As^3+^, As^5+^, Hg^2+^, Cd^2+^, Pb^2+^, Cr^3+^, Cr^6+^, and Cu^2+^. The materials demonstrated high stability during continuous-flow column treatment, and the adsorbents could be regenerated with a gentle acidic treatment.

**Table 4:** Summary of one MOF for removal of different contaminants.

Catalyst	Removal Species (Adsorption Capacity, Time for Equilibrium)	Year	Ref.
MTV-MOF	Hg^2+^, Pb^2+^, and Tl+; Pyronin Y, Auramine O, brilliant green, and methylene blue, 0–48 h	2019	[16]
Zr-MOF	Methylene blue (169 mg/g) Lead ions (100 mg/g) Cadmium ions (37 mg/g)	2021	[125]
CaFu MOF	Imidacloprid (467.23 mg/g, 150 min) Cd^2+^ ions (781.2 mg/g, 40 min)	2021	[126]
Cu-MOFs/CMFP	Methylene blue, malachite green, and rhodamine B; Pb^2+^ and Cd^2+^	2021	[132]
ZnO-NP@Zn-MOF-74	Cu (106.27 mg/g, 2 h) Tetracycline (137.17 mg/g, 2 h)	2021	[17]
ZIF8/MWCNT	Phosphate (188.5 mg/g, 12 h) Acetaminophen (0.51 mol/g, 12 h) Triclosan (0.35 mol/g, 12 h)	2021	[128]
2D-MOF@C@FeO	Microplastic (100% removal, 60 min) Methylene blue (100% removal, 60 min)	2023	[129]
MOF-5/COF	Auramine O (17.95 mg/g, 10 min) RhodamineB (16.18 mg/g, 10 min)	2020	[30]
*apt*-NiCoFe-MOF-74	TC (97.4 mg/g)	2024	[9]
	OTC (105.2 mg/g, 10 min)		
	CTC (115.6 mg/g, 10 min)		
	DOX (114.7 mg/g, 10 min)		
Eu^3+^-MOF	Malachite green (97.64%, 120 min),	2024	[15]
	Leuco-malachite green		
Zr-MOF	Pb^2+^ (715.2 mg/g, 30 min) Hg^2+^ (862.7 mg/g, 30 min) Cd^2+^ (450.5 mg/g, 30 min)	2024	[130]
NH_2_-MIL-101-Fe, and MOF-808-EDTA	Hg^2+^(272.7 mg/g) As^3+^ (151.29 mg/g) Mn^2+^ (125.9 mg/g)	2024	[77]
CeO_2_@UiO-66	As^3+^, As^5+^, Cd^2+^, Cr^3+^, Cr^6+^, Cu^2+^, Pb^2+^, and Hg^2+^	2020	[131]

MOFs offer a promising solution for the simultaneous removal of multiple heavy metal ions from wastewater due to their selective adsorption, high capacity and efficiency, tunable structures, and environmental compatibility. These properties make MOFs an attractive option for addressing the challenges of mixed heavy metal contamination in wastewater, contributing to environmental protection and sustainable development. As many studies focus on the adsorption of multiple heavy metals, further discussion on this topic is beyond the scope of this review.

#### 4.2.2. Detection of Different Contaminants

Simultaneous detection of multiple contaminants in wastewater is crucial for effective environmental monitoring and pollution control [38,50]. Traditional sensing methods often require multiple sensors or complex analytical equipment, which can be time-consuming and costly [20]. However, MOF-based sensors offer a promising alternative by integrating multiple detection functionalities within a single material. Researchers are focused on enhancing the performance of MOF-based sensors in various aspects, including ease of fabrication, high sensitivity and selectivity, versatility, tunability, and environmental compatibility. A summary of MOFs used for the detection of different contaminants in recent years is shown in Table 5.

Insecticides are widely used in agriculture, forestry, and urban pest control to manage pests and protect crops [133]. However, their widespread use has led to significant environmental pollution [39,134]. Developing efficient insecticide detection methods is therefore essential for environmental protection. Zhou’s group [38] used a template method to synthesize Cu/Co-MOF with a hollow structure, which, when combined with a graphite-like carbon nitride nanosheet (g-C_3_N_4_) and luminol, formed a dual-signal luminescent sensor for the simultaneous detection of the insecticides malathion and acetamiprid. The aptamers for malathion and acetamiprid were loaded onto Cu/Co-MOF and AuNPs/g-C_3_N_4_, respectively. The hollow structure of Cu/Co-MOF significantly reduced electron mass transfer resistance, enhancing the material’s conductivity. The sensor demonstrated a linear range of 0.1 μM to 0.1 pM, with LODs of 0.015 pM for malathion and 0.018 pM for acetamiprid. In another study [39], the same group synthesized a Ce(III, IV)-MOF composite with hionine (Thi) and ferrocene (Fc) probes which was used to simultaneously detect pesticides like malathion and chlorpyrifos.

Catechol (CC) and hydroquinone (HQ) are common phenolic compounds used in various industries. Their environmental pollution has raised significant concerns [135,136]. Huang et al. [50] prepared a composite of Ce-MOF and carbon nanotubes (CNTs), followed by post-treatment with NaOH/H_2_O_2_. The Ce-MOF’s central atom, which has two valences, along with the electron-conducting CNTs, enabled the sensor to effectively discriminate between CC and HQ. The sensor exhibited excellent performance with LODs of 3.5 μM for CC and 5.3 μM for HQ. Similarly, Zheng et al. [137] synthesized a CoNi-MOF and graphene oxide (GO) composite for the electrochemical detection of CC and HQ. The sensor demonstrated a wide linear range (0.1–100 μM), LODs of 0.04 and 0.03 μM for HQ and CC, respectively, and an excellent anti-interference ability.

Heavy metal contamination in wastewater poses significant risks to ecosystems and human health. Consequently, many MOF-based materials have been developed for the simultaneous detection of heavy metal ions. For example, Hou’s group [138] synthesized a CoZn-MOF composite with conductive carbon paper and reduced graphene oxide (rGO) to create a sensing electrode (CP-rGO-CoZn-MOF) for the simultaneous detection of Cd^2+^ and Pb^2+^ ions. The sensor achieved LODs of 0.565 nM for Cd^2+^ and 0.588 nM for Pb^2+^. Similarly, Ha et al. [139] fabricated an Yb-MOF composite, which was drop-cast onto a glassy carbon electrode to detect Cd^2+^ and Pb^2+^ ions with LODs of 3.0 ppb and 1.6 ppb, respectively. Additionally, Cu-MOF-based electrodes have been used to detect Tl^+^ and Hg^2+^ ions in electrochemical applications [140].

**Table 5:** Summary of MOFs for detection of different contaminants.

Catalyst	Detection Method	Detection Species (LOD)	Year	Ref.
Cu/Co-MOF	Electrochemical	Malathion (0.015 pM) Acetamiprid (0.018 pM)	2021	[38]
Ce(III, IV)-MOF	Electroluminescence	Malathion (0.038 pM) Chlorpyrifos (0.045 pM)	2022	[39]
Ce-MOF/CNTs	Electrochemical	Catechol (3.5 μM) Hydroquinone (5.3 μM)	2021	[50]
CoNi-MOF/GO	Electrochemical	Catechol (0.03 μM) Hydroquinone (0.05 μM)	2023	[137]
CP-rGO-CoZn-MOF	Electrochemical	Cd^2+^ (0.565 nM) Pb^2+^ (0.588 nM)	2022	[138]
Yb-MOF	Electrochemical	Cd^2+^ (3.0 ppb) Pb^2+^ (1.6 ppb)	2021	[139]
Cu-MOF	Electrochemical	Tl^+^ (0.11 ppb) Hg^2+^ (0.17 ppb)	2020	[140]

In most studies on simultaneous detection using MOFs, the electrochemical method has been the focus, primarily due to its high sensitivity compared to other detection methods. This is particularly advantageous when MOFs are incorporated into electrodes; however, the trace amounts on the electrode surface limit adsorption and other functions to some extent. While other detection methods exist, the electrochemical approach remains the most commonly used for MOF-based sensors in contaminant detection due to its sensitivity and efficiency.

## 5. Conclusions, Outlooks, and Recommendations

### 5.1. Conclusions

MOFs have demonstrated significant potential in wastewater treatment because of their high porosity and specific adsorption sites which enable efficient removal of a wide range of contaminants, including both organic and inorganic pollutants. Moreover, MOFs can be functionalized with specific ligands or metal ions to enhance their selectivity for target contaminants, allowing for the targeted removal of pollutants and reducing the need for extensive and costly treatment processes.

The tunable properties of MOFs make them ideal candidates for sensor development. By incorporating specific metal ions or ligands, MOF-based sensors can interact with target reactants, operating through various detection mechanisms such as fluorescence quenching, colorimetric changes, or electrical conductivity alterations. These mechanisms can be customized to meet the specific needs of different detection applications, providing a versatile platform for monitoring a wide range of contaminants.

In addition to adsorption and sensing, MOFs can also be designed to include catalytic metal ions or clusters that facilitate the degradation of contaminants. Their large surface area and high porosity provide numerous catalytic sites, enhancing the efficiency of these processes. The tunability of MOFs further allows for the incorporation of specific ligands that improve selectivity, making them highly effective for targeted contaminant degradation.

The versatility of MOFs enables the creation of multifunctional platforms capable of addressing multiple contaminants simultaneously. This reduces the need for multiple treatment steps, streamlining the process and leading to cost savings. By leveraging the unique properties and functionalities of MOFs, we can develop more effective, efficient, and sustainable wastewater treatment processes, contributing to environmental protection and sustainability. However, it should be noted that it is challenging for multifunctional MOF materials to outperform single-functional MOF materials due to potential interference among the functions. Therefore, it is considered satisfactory if the individual functions do not degrade in multifunctional materials.

### 5.2. Future Outlooks

There are several efforts that need to be made to fully harness the potential of multifunctional MOFs in the treatment of contaminants in an aquatic environment: (1) Continued efforts are needed to design and synthesize MOFs with specific functionalities that target different contaminants in wastewater. This includes tuning the pore size, shape, and chemistry of the MOFs to optimize their adsorption and catalytic properties. (2) A deeper understanding of the mechanisms underlying the adsorption and catalytic processes in MOFs is needed to optimize their performance. This includes studying the interactions between MOFs and contaminants, as well as the effects of environmental conditions on these interactions.

So that multifunctional MOF materials can be used in industrial applications for real wastewater treatment, several efforts need to be made: (1) The economic feasibility of producing and using MOFs on a large scale needs to be improved, and developing cost-effective methods should be deeply studied. (2) The stability and durability should be greatly enhanced, because in real wastewater, many harsh conditions are common, for example, high temperatures, acidic or alkaline pH levels, and the presence of oxidizing agents. (3) The environmental impact should be evaluated because there is the potential release of metal ions or other contaminants from the MOFs.

Overall, the successful application of multifunctional MOFs in wastewater treatment will require a concerted effort across multiple disciplines and research areas. By addressing these key challenges, we can harness the unique properties of MOFs to develop more effective, efficient, and sustainable contaminant treatment in an aquatic environment. In addition, the development of MOFs can be enhanced by artificial intelligence (AI).

### 5.3. Future Recommendation

A significant number of studies have focused on the application of MOFs in treating contaminants in aquatic environments. However, research on multifunctional MOFs remains scarce.

In fundamental research on MOF materials used in wastewater treatment, we believe that the intelligent design of MOFs represents a crucial research direction. Attention should be given to MOFs that exhibit high efficiency in monitoring and eliminating contaminants, or even converting them into high-value-added products. Furthermore, a broader range of contaminants should be considered, such as microplastics and some fluorine compounds in aquatic environments. To advance the industrial application of MOFs in real-world wastewater treatment, efforts should be directed toward reducing costs, enhancing stability (including hydrothermal and chemical stability), increasing reusability, and developing production methods that are readily industrializable.

Utilizing AI algorithms for optimization and simulation techniques, MOF materials with specific molecular structures and chemical functionalities can be designed. These materials can efficiently adsorb, detect, and degrade targeted pollutants. Additionally, integrating MOFs with AI technology enables the construction of an intelligent water pollution treatment system that integrates detection, adsorption, and degradation into one system. This system could achieve precise identification, efficient adsorption, and complete degradation of pollutants, providing a comprehensive and efficient solution for water pollution treatment. In summary, the combination of MOFs and AI technology will bring revolutionary changes to the detection, adsorption, and degradation treatment of water pollutants.

## Data Availability

No new data were created or analyzed in this study. Data sharing is not applicable to this article.
